# PACAP-Deficient Mice Exhibit Light Parameter–Dependent Abnormalities on Nonvisual Photoreception and Early Activity Onset

**DOI:** 10.1371/journal.pone.0009286

**Published:** 2010-02-18

**Authors:** Chihiro Kawaguchi, Yasushi Isojima, Norihito Shintani, Michiyoshi Hatanaka, Xiaohong Guo, Nobuaki Okumura, Katsuya Nagai, Hitoshi Hashimoto, Akemichi Baba

**Affiliations:** 1 Graduate School of Pharmaceutical Sciences, Osaka University, Osaka, Japan; 2 Institute for Protein Research, Osaka University, Osaka, Japan; 3 Genomic Science Center, RIKEN, Yokohama, Japan; 4 The Osaka-Hamamatsu Joint Research Center for Child Mental Development, Osaka University, Osaka, Japan; 5 United Graduate School of Child Development, Osaka University, Kanazawa University, and Hamamatsu University School of Medicine, Osaka, Japan; Tokyo Institute of Psychiatry, Japan

## Abstract

**Background:**

The photopigment melanopsin has been suggested to act as a dominant photoreceptor in nonvisual photoreception including resetting of the circadian clock (entrainment), direct tuning or masking of vital status (activity, sleep/wake cycles, etc.), and the pupillary light reflex (PLR). Pituitary adenylate cyclase-activating polypeptide (PACAP) is exclusively coexpressed with melanopsin in a small subset of retinal ganglion cells and is predicted to be involved extensively in these responses; however, there were inconsistencies in the previous reports, and its functional role has not been well understood.

**Methodology/Principal Findings:**

Here we show that PACAP-deficient mice exhibited severe dysfunctions of entrainment in a time-dependent manner. The abnormalities in the mutant mice were intensity-dependent in phase delay and duration-dependent in phase advance. The knockout mice also displayed blunted masking, which was dependent on lighting conditions, but not completely lost. The dysfunctions of masking in the mutant mice were recovered by infusion of PACAP-38. By contrast, these mutant mice show a normal PLR. We examined the retinal morphology and innervations in the mutant mice, and no apparent changes were observed in melanopsin-immunoreactive cells. These data suggest that the dysfunctions of entrainment and masking were caused by the loss of PACAP, not by the loss of light input itself. Moreover, PACAP-deficient mice express an unusually early onset of activities, from approximately four hours before the dark period, without influencing the phase of the endogenous circadian clock.

**Conclusions/Significance:**

Although some groups including us reported the abnormalities in photic entrainments in PACAP- and PAC_1_-knockout mice, there were inconsistencies in their results [1], [2], [3], [4]. The time-dependent dysfunctions of photic entrainment in the PACAP-knockout mice described in this paper can integrate the incompatible data in previous reports. The recovery of impaired masking by infusion of PACAP-38 in the mutant mice is the first direct evidence of the relationship between PACAP and masking. These results indicate that PACAP regulates particular nonvisual light responses by conveying parametric light information—that is, intensity and duration. The “early-bird” phenotype in the mutant mice originally reported in this paper supposed that PACAP also has a critical role in daily behavioral patterns, especially during the light-to-dark transition period.

## Introduction

Behavioral and physiological adaptations to external day/night cycles are regulated by a variety of environmental cues. Among these cues, light is considered to be the most universal and efficient [5]. Light induces phase-shifting of the circadian clock (entrainment), direct tuning or masking of vital status (activity, sleep/wake cycles, hormone secretion), and pupil constriction, so that the adaptations can be achieved [6], [7], [8], [9]. These ‘nonvisual’ light responses have been suggested to be predominantly mediated by the novel photopigment melanopsin [10], [11].

Pituitary adenylate cyclase-activating polypeptide (PACAP) belongs to the vasoactive intestinal polypeptide (VIP)/glucagons/secretin family and plays pleiotropic roles as a neurotransmitter, neuromodulator and neurotrophic factor [12]. In the retina, PACAP is exclusively expressed with melanopsin [13] in a small subset of retinal ganglion cells [14], [15]. PACAP-containing RGCs innervate widespread brain areas including the suprachiasmatic nucleus (SCN: the master circadian clock in mammals), paraventricular zone (the area involved in masking) and olivary pretectal tract (OPT: the crucial node in the pupillary reflex circuit) [16]. Previous studies using transgenic mice [1], [2], [3], [4] or SCN slice cultures [17], [18] suggested the involvement of PACAP signaling in light- and/or glutamate (a major light transmitter)**–**induced phase shifts. However, varieties and inconsistencies among these results have complicated our understanding of the role of PACAP: slice cultures analyses suggest that PACAP is both an inducer and a modulator of phase shifts, because PACAP at nanomolar concentrations is alone sufficient to induce phase shifts [17], yet higher concentrations of PACAP enhance or repress glutamate-induced phase delay or advance, respectively [18]. Mice lacking PACAP (*Adcyap1* ^−/−^) or the PACAP**–**specific receptor PAC_1_ (PAC_1_^−/−^) show a common impairment of phase advance, but intricate differences in phase delay [1], [2], [3], [4]. In addition, relationships between PACAP and other nonvisual light responses were reported [2], [4], [19], but discrepancies in their results were also found.

To clarify the functional roles of intrinsic PACAP in nonvisual photoreception, we examined entrainment, masking and the pupillary light reflex (PLR) in *Adcyap1* ^−/−^ mice. The present study indicates that PACAP controls particular nonvisual light responses; that is, entrainment and masking. In mediating these two responses, we suggest that PACAP transmits light information such as irradiances and durations. Additionally, PACAP has been shown to affect daily activity patterns under a light/dark (LD) cycle through one or more non-entrainment mechanisms.

## Results

### Entrainment

**Figure 1:**
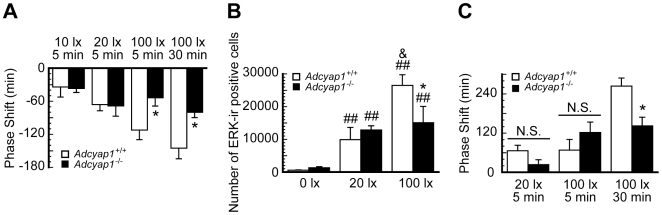
Impaired light-induced behavioral phase shift in *Adcyap1* ^−/−^ mice. (**A**) Quantification of light-induced phase delay at CT15 (n = 5–7 for 5 min, n = 4–5 for 30 min). (**B**) Numbers of phosphorylated ERK-immunoreactive (pERK-Ir.) cells in the SCN (n = 4–7). (**C**) Quantification of light-induced phase advance at CT21 (n = 4–5). Values denote means ± SEM. ^*^ *p*<0.05 versus *Adcyap1* ^+/+^ mice at 100lx, ^##^ *p*<0.01 versus each genotype at 0lx, ^&^ *p*<0.05 versus *Adcyap1* ^+/+^ mice at 20lx; Mann-Whitney *U* test followed by Kruskal-Wallis Test.

As described previously [1], *Adcyap1* ^−/−^ mice could synchronize with a 12 hr light:12 hr dark LD cycle (12L∶12D) and retained behavioral periodicity under constant dark (DD) conditions (**Fig. S1A**). We tested the entrainment function of *Adcyap1* ^−/−^ mice by exposing them to a short light pulse at the indicated circadian time (CT). A dim light pulse at early subjective night (CT15) (10 or 20lx, 5 min) induced phase delay of locomotor activity rhythms in *Adcyap1* ^−/−^ mice, similar in magnitude to that in *Adcyap1* ^+/+^ mice, but a brighter pulse (100lx, 5 min) failed to produce any further shifting in the mutants (**Fig. 1A** and **Fig. S1A**). This impaired phase delay was not restored, even when the duration of light stimulation was prolonged to 30 min (**Fig. 1A** and **Fig. S1A**). Consistent with the behavioral deficits, light–induced phosphorylation of extracellular signal-regulated kinase 1/2 (ERK) in the SCN, which is suggested to be a highly sensitive light detector and a determinant of the magnitude of phase delay [20], reached a ceiling at 20lx preventing further phosphorylation in *Adcyap1* ^−/−^ mice at 100lx (**Fig. 1B** and **Fig. S1B**). Unlike phase delay, phase advance elicited by a light pulse at late subjective night (5 min, CT21) was not altered in *Adcyap1* ^−/−^ mice, either at 20 or 100lx (**Fig. 1C**). However, *Adcyap1* ^−/−^ mice showed a significant smaller phase advance when the light duration was prolonged to 30 min (**Fig. 1C**).

### Masking Responses

**Figure 2:**
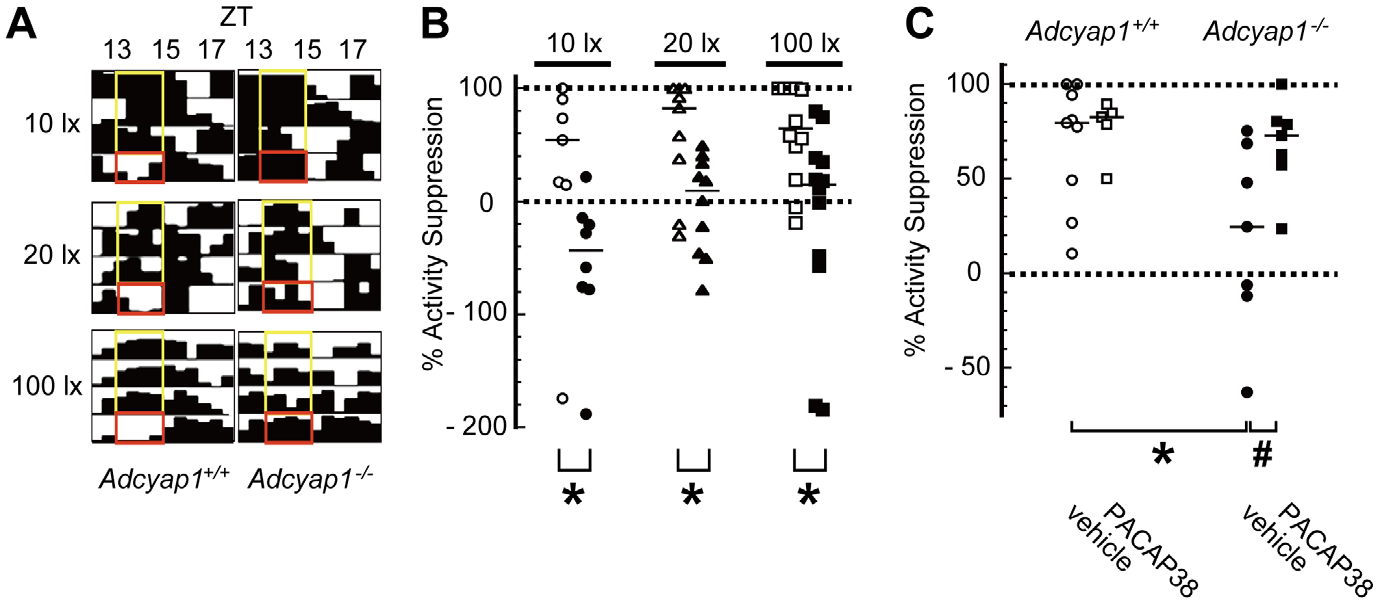
Impaired activity masking in *Adcyap1* ^−/−^ mice. (**A**) Wheel-running records for ZT12–18. Actograms are magnified to show direct responses to a two-hour light pulse at ZT13–15 on test days (red boxes) and no pulse during a comparable time on the three previous nights (yellow boxes). (**B**) Quantification of % activity suppression during the first hour (ZT13–14). The solid bar in each group denotes the median (n = 7–12). Open symbols: *Adcyap1* ^+/+^, closed symbols: *Adcyap1* ^−/−^. (**C**) The effect of PACAP supplementation (20 pmol) on masking responses 30 min before photic stimulation at ZT13 (100lx). The solid bar in each group denotes the median (n = 7–8). ^*^ *p*<0.05 versus *Adcyap1* ^+/+^ in each intensity of light or vehicle-treated *Adcyap1* ^+/+^, ^#^ *P*<0.05 versus vehicle-treated *Adcyap1* ^−/−^; Mann-Whitney *U* test.

Masking modulates vital status with time by detecting ambient luminance [8], [9]; for example, nocturnal animals are active when the surroundings are dark (<∼0.2lx; positive masking) and passive in a brighter milieu (>∼10lx; negative masking) [21]. Such reactions occur without influencing the underlying circadian clock. We assessed masking by quantifying the amount of activity induced by a two-hour light pulse during early night, a stimulus that is specifically designed for the estimation of masking. *Adcyap1* ^+/+^ mice exhibited clear light-induced locomotor suppression (negative masking) at each light intensity (**Fig. 2A**). Because this negative masking in *Adcyap1* ^+/+^ mice was predominantly occurred in the first an hour during a two-hour-light exposure, we compared suppression rates during the first hour only between two genotypes. *Adcyap1* ^+/+^ mice showed more than 50% suppression of activity rate at all of the light intensities investigated, but this negative masking was significantly impaired in *Adcyap1* ^−/−^ mice, at all intensities tested, and several mutants paradoxically became active (paradoxical positive masking), especially at the lowest light intensity (**Fig. 2A, B**). These abnormalities were ameliorated by intracerebroventricular administration of PACAP38 (20 pmol) 30 min before exposure to 100lx light, whereas this concentration of PACAP38 had no effect on the amount of activity in *Adcyap1* ^+/+^ mice (**Fig. 2C**).

### PLR

**Figure 3:**
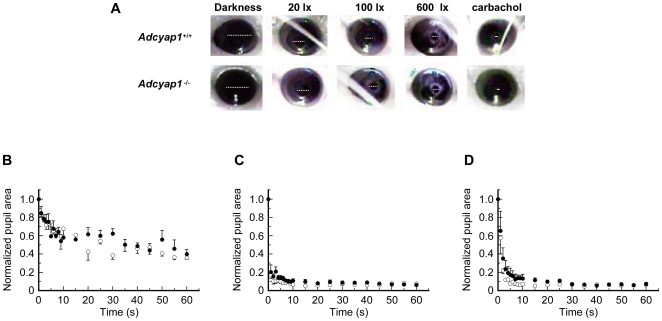
PLR in *Adcyap1* ^−/−^ mice. (**A**) Photographs of pupillary constriction 1 min post-irradiation. Darkness: scotopic conditions; carlbachol: 1 min after topical instillation of carlbachol under dark conditions. White broken lines indicate pupillary diameters. (**B–D**) Time courses of pupillary miosis during 1 min post-irradiation [(**B**) 20lx; (**C**) 100lx; (**D**) 600lx]. The graphs indicate normalized pupil area relative to time 0. Values denote means ± SEM (n = 4–7). No statistical differences were seen in all light intensities (two–way repeated-measures ANOVA).

There were no differences in pupil sizes under scotopic conditions between genotypes (*Adcyap1* ^−/−^, 1.06±0.18 mm^2^; *Adcyap1* ^+/+^, 0.98±0.10 mm^2^). A PLR was detected in *Adcyap1* ^−/−^mice in response to 460–490 nm blue light, and this was similar in magnitude and time course to that detected in *Adcyap1* ^+/+^ mice at all light intensities (two–way repeated-measures ANOVA, **Fig. 3B–D**). Additionally, the minimal pupil areas attained by *Adcyap1* ^−/−^ mice in 1 min of steady light were comparable to those of *Adcyap1* ^+/+^ mice (**Fig. 3A**).

### Retinal Morphology, Innervation and PACAP-Related Gene Expression

**Figure 4:**
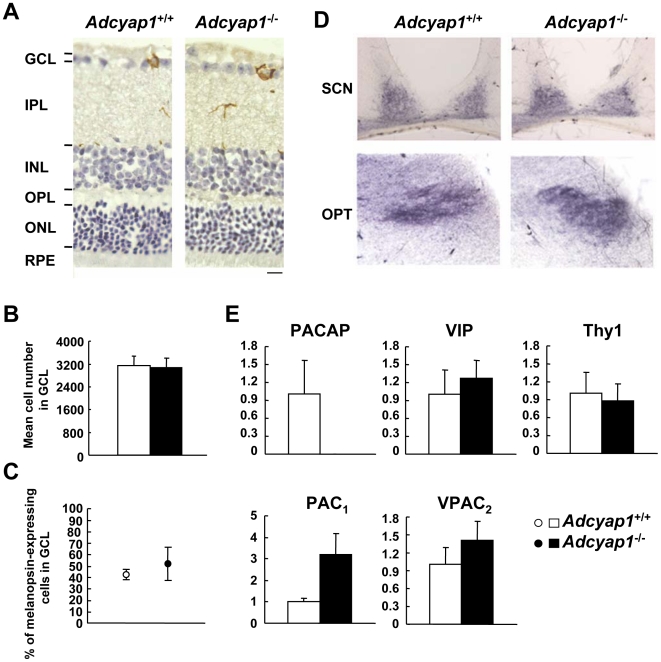
Retinal morphology, innervation and expression of PACAP-related genes in the eye. (**A**) Immunohistochemical analysis of melanopsin localization (brown) in the retina. Slices were counterstained with hematoxylin. GCL: ganglion cell layer; IPL: inner plexiform layer; INL: inner nuclear layer; OPL: outer plexiform layer; ONL: outer nuclear layer; RPE: retinal pigment epithelium. Scale bar = 10 µm. (**B**) Mean cell number in the GCL (n = 4). (**C**) Percentage of melanopsin-expressing cells in the GCL (n = 4). (**D**) Bright-field photomicrographs of retinal innervation to the SCN and the OPT, stained purple. (**E**) Expression levels of PACAP-related genes in the whole eye (n = 3–4). Means ± SEM are presented.

PACAP exerts neurotrophic and neuroprotective effects and modulates cellular differentiation and survival [12]. In the retina, PACAP promotes retinal development through its specific receptor, PAC_1_ [22], [23]. Thus, we examined whether there was any remodeling in terms of morphology, neuronal projections and PACAP-related gene expression, in the retinas of *Adcyap1* ^−/−^ mice. *Adcyap1* ^−/−^ mice showed retention of somatic and dendritic/axonal layers, and melanopsin expression in the ganglion cell layer (**Fig. 4A–C**). Anterograde tracing with the use of horseradish peroxidase–labeled wheat germ agglutinin disclosed normal projections from RGCs to the SCN or OPT in the mutants (**Fig. 4D**). Gene expression analysis (**Fig. 4E**) indicated that there were no changes in the expression of VIP (a peptide that has approximately 70% amino acid sequence identity with PACAP), VPAC_2_ (VIP receptor subtype 2: a receptor for both PACAP and VIP), and Thy1 (thymus cell antigen 1, theta: a marker of the retinal ganglion cell layer) [24]. PAC_1_ expression showed a tendency to increase in *Adcyap1* ^−/−^ mice, but this difference did not reach statistical significance (*P* = 0.10). We confirmed the complete loss of PACAP mRNA in *Adcyap1* ^−/−^ eye.

### Daily Activity Rhythms

**Figure 5:**
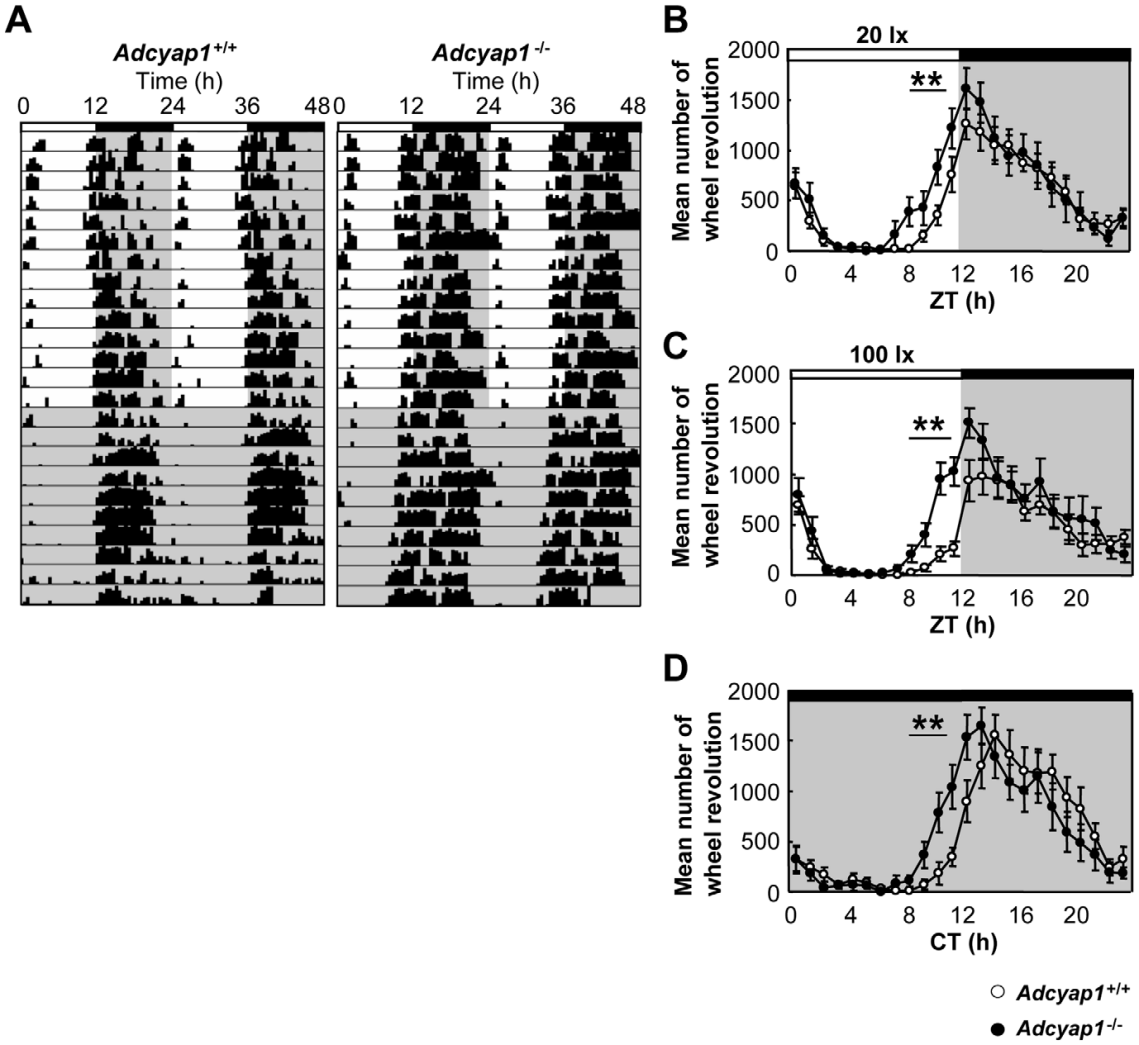
Early onset of activities in *Adcyap1* ^−/−^ mice. (**A**) Representative double-plotted actogram in LD cycles (light phase 20lx) and subsequent DD conditions. Dark phases are shaded. (**B–C**) Quantification of wheel-running activities in the LD cycles with a 20lx (**B**) or 100lx (**C**) light phase (n = 12). (**D**) Quantification of wheel-running activities in subsequent DD conditions (after 20lx LD cycle; n = 12). Values denote means ± SEM ^**^ *p*<0.01 versus *Adcyap1* ^+/+^ during ZT/CT 8–11, two–way repeated-measures ANOVA.

**Figure 6:**
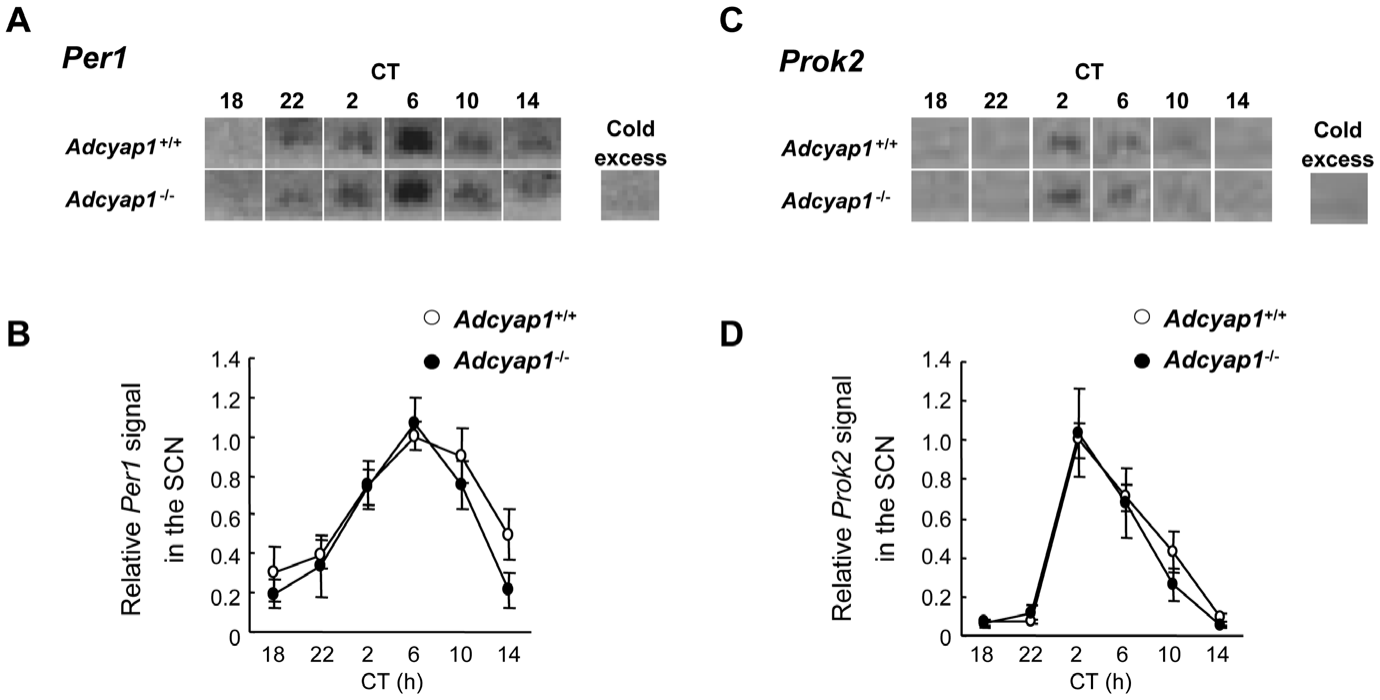
Circadian fluctuations of *Per1* and *Prok2* in the SCN under constant dark conditions. Photomicrographs (**A, C**) represent mRNA expression in the SCN by *in situ* hybridization and line charts (**B, D**) show the expression levels of *Per1* (**A, B**; n = 3–6) and *Prok2* (**C, D**; n = 4–6). As a control, *in situ* hybridization was performed in the presence of a 100–fold cold excess of unlabeled probes.

Adaptation of daily activity rhythms to environmental LD cycles is reported to be attained by harmonization with entrainment and masking [7]. We tested the effects of the deficits in entrainment and masking found in *Adcyap1* ^−/−^ mice on their behavioral adaptation. Under LD cycles with a 20 or 100lx light phase, *Adcyap1* ^−/−^ mice showed robust behavioral rhythms nearly identical to those in *Adcyap1* ^+/+^ mice (**Fig. 5A, B**); however the mutants unexpectedly exhibited an earlier onset of activities, about four hours before the dark phase, but showed a normal offset latencies after the dark phase (**Fig. 5A, B**). These aberrant activities were still observed in subsequent DD conditions (**Fig. 5A, C**). We hypothesized that this early onset of activity was ascribable to molecular clock disturbances with a positive phase angle; therefore, we examined the circadian fluctuation of *Period1* (*Per1*: a major clock gene that determines the free-running period) [25] and *Prokineticin2* (*Prok2*: a clock-controlled gene that suppresses day-time activity) [26] transcripts in the SCN. *Adcyap1* ^+/+^ mice showed remarkable circadian fluctuations in both genes with the same peak times (**Fig. 6**), as reported previously [25], [26]. Inconsistent with our hypothesis, *Adcyap1* ^−/−^ mice showed an almost all identical phase angle and waveform to those of *Adcyap1* ^+/+^ mice. Additionally, the free-running period in DD conditions was not altered in *Adcyap1* ^−/−^ mice (*Adcyap1* ^−/−^, 23.7±0.09 hours, n = 12; *Adcyap1* ^+/+^, 23.8±0.08 hours, n = 12).

## Discussion

The present study shows that PACAP regulates particular nonvisual light responses in time–, irradiance– and duration–dependent manners, and that a PACAP deficit leads to an unusually early onset of activities without changing the phase of the molecular clock. Our findings suggest that PACAP transmits parametric light information, such as fluctuation of light intensities or durations that occur continuously under natural conditions, and that PACAP contributes to formation of daily activity patterns, notably at dusk, by a non–clock entrainment system.

Previous anatomical studies of PACAP/melanopsin-containing retinal innervation suggested roles of PACAP/melanopsin in a broad variety of nonvisual photoreception [16], [27]. In particular, the SCN and OPT are major target areas of PACAP/melanopsin–positive RGCs. Unlike the anatomy-based prediction that PACAP is especially important for functions of these major target areas, *Adcyap1* ^−/−^ mice exhibited abnormalities in entrainment and masking, but normal PLRs. This could be attributed to differences in the number of melanopsin-expressing RGCs projecting to the area involved in each of these functions. Melanopsin-expressing RGCs represent a large majority of the retinal projections to the entrainment center, the SCN, and the area underlying masking, the ventral subparaventricular zone (approximately 70% of the total retinal input), but only a minority of the retinal projection to the critical node of PLR circuit OPT (about 10%) [27].

The circadian clock is reset daily by environmental timing cues such as dusk and dawn; these stimuli induce phase delay or advance of circadian rhythms, respectively. In this study, we found that intrinsic PACAP exquisitely controls both phase delay and advance in time-, irradiance-, and duration-dependent manners, not merely inducing phase shifts. At early subjective night, PACAP appears to transmit information regarding light of a high intensity, because *Adcyap1* ^−/−^ mice lost the ability to exhibit further phase delay shifting and ERK phosphorylation in the presence of bright light (100lx). This suggestion is supported by previous *in vitro* studies showing that PACAP potentiates glutamatergic signaling at early subjective night [28] and glutamate-induced phase delay of the firing rhythms in the SCN [18]. At brighter irradiances, light duration seems unlikely to be a critical factor for phase delay, since *Adcyap1* ^+/+^ mice show maximal amplitudes of phase delay after a short (5 min) light pulse, consistent with a previous report [29]. This irradiance-dependent profile of PACAP could explain previous discrepancies in *Adcyap1* ^−/−^ mice, such as a normal phase delay at 20lx (30 min) [1], but an impaired phase delay at 50 or 500lx (15 min) [2]. Also our results were consistent with the results in the PAC_1_-dificient (PAC_1_^−/−^) mice showed blunted phase delay at 300lx (30 min) [4]. Unlike phase delay, PACAP seems to be involved in light-induced phase advance at late subjective night, in a duration–dependent manner, because *Adcyap1* ^−/−^ mice exhibited a significantly impaired phase advance in response to a 30 min light pulse (with 100lx in this study, and 20lx in our previous one [1]), but not to a 5 min light pulse, regardless of the irradiances. This finding is consistent with previous reports of deficits in the phase advances of mice lacking PACAP signaling [2], [3], [4]. These researchers used a light duration longer than 5 min (PAC_1_^−/−^ mice: 30 min, ≥300lx; *Adcyap1* ^−/−^ mice: 15 min, 500lx). Therefore, intrinsic PACAP signaling is supposed to participate in phase advance events, upon and after 5 min of light exposure. By contrast, no *in vitro* studies agree with the present finding, because PACAP inhibits glutamate-induced phase advances of firing rhythms at higher concentrations [18], but produces a phase advance at lower concentrations [17]. This might be attributed to differences between the physiological and pharmacological concentrations of PACAP. The varied effects of PACAP in early- and late-subjective night are supposed to be caused by the circadian regulations in signaling cascade of glutamate and PACAP in the SCN, but further analyses are necessary.

Although *Adcyap1* ^−/−^ mice exhibited severe deficits in masking, they incompletely detected luminance, as their suppression rates only slightly increased in increasing light intensities. These results are consistent with the recent reports using PAC_1_^−/−^ mice [4]. These incomplete responses were observed even when *Adcyap1* ^−/−^ mice were exposed to bright light (100lx; an intensity sufficient to induce maximal negative masking) [21], but were completely rescued by supplementation with PACAP38. Importantly, PACAP38 administration did not exert any effects on the basal activity levels of *Adcyap1* ^+/+^ mice. Therefore, these data indicate that the loss of PACAP is the primary cause of disturbed negative masking and that PACAP is essential to maintain the photic sensitivity of this response. These results, taken together with the results on entrainment, suggest that PACAP-mediated transmission of parametric light information is prerequisite for both entrainment and masking, but that PACAP regulates each function in a distinct manner. Masking disturbances in *Adcyap1^−/−^* mice could be also related to melanopsin-mediated phototransduction, because melanopsin is a photoreceptor that is exclusively colocalized in PACAP-expressing ganglion cells in the retina [13], and because melanopsin is suggested to dominantly mediate the transduction of photic information for negative masking [21], [30]. Unexpectedly, several *Adcyap1^−/−^* mice showed a locomotor increase in response to all three intensities of light. This ‘paradoxical’ positive masking might occur due to diminished negative masking as a result of disturbances in melanopsin/PACAP signaling, leaving positive masking, which is driven by rod/cone cells [31], [32]. Since there were no remarkable changes in retinal structures or PLR in *Adcyap1^−/−^* mice, their dysfunctions of entrainment and masking are suggested to arise not from light input *per se*, but from loss of PACAP in a neuronal circuit after the retina.

*Adcyap1^−/−^* mice showed early onset of activities under LD cycles and in DD conditions, without changing the phase of the circadian clock, indicating that there is no relation between predark activities and molecular clock phase. There remains the possibility that impaired entrainment in mutants is causal for predark activities, but this idea seems unlikely to be supported because phase of the molecular clocks are almost identical between the two genotypes, and the daily behavioral patterns were also identical with the predark periods being the only exception. Another possibility is aberrant masking. Masking, especially positive masking, has been suggested to have a circadian phase dependency. Several lines of evidence indicate that maximal increases in positive masking occur around the onset of the night phase, even in constant conditions [33]. Other lines of evidence indicate a strong correlation of paradoxical positive masking with light/dark transition periods [7]. In addition, several transgenic mice with masking disturbances often exhibited unusual activities during the transitions between light and dark [21], [34], [35]. Among these mutant mice, the phenotypes of melanopsin^−/−^ mice strongly resemble those of *Adcyap1^−/−^* mice in terms of diminished negative masking and predark activities in both LD and DD conditions [21], [36]. Thus, these previous reports seem to agree with the idea that deficits in negative masking contribute to early onset of activities in *Adcyap1^−/−^* mice. Further analyses are warranted to investigate the underlying mechanism.

## Materials and Methods

### Ethics Statement

All animal care and handling procedures were approved by the Institutional Animal Care and Use Committee of Osaka University, the Guiding Principles for the Care and follow the United States National Institutes of Health Guide for the Care and Use of Laboratory Animals.

### Mice

The generation of *Adcyap1* ^−/−^ mice by gene-targeting has been reported previously [37]. The null mutation was backcrossed ten times onto a CD1 (ICR) genetic background. We should note that each mouse was kept in the insulated box. The boxes were equipped with an electric bulb for illumination and a measuring device (a far-infrared apparatus or a running wheel) (Bio-Medica, Japan) to monitor the behavioral activities of the mouse. Thus all experiments described below were performed without irritating mice by moving the cages and without affected by the neighbor mice.

### Entrainment

Light-induced phase shifts of locomotor activity rhythms were examined as described [1]. Briefly, mice were transferred to constant dark (DD) conditions after being entrained to a 24-hour LD (12L∶12D) cycle, with monitoring of their locomotor activity by far-infrared apparatuses. After more than eight days in DD conditions, animals were exposed to a white light pulse of the indicated intensities at circadian time (CT)15 or CT21, and their behavioral rhythms were further recorded. The phase shifts were calculated based on the distance between two regression lines drawn from the onset of activity before and after the light pulse as described in [38], supported by the custom-made period-detection software based on autocorrelation [39].

### Masking Responses

Mice were entrained to a 24-hour LD (12L∶12D) cycle, with monitoring of their locomotor activity by running wheels. A light pulse was given to the animals for 2 hours from Zeitgeber time (ZT)13. The percentage of activity suppression during the first hour after the stimulus was calculated using the following formula [30]: % activity suppression = (B–A)/B * 100, where A is the amount of activity during the first hour after ZT13 on the day on which light stimulations were given, and B is the average activity level during a comparable time period during the previous three days.

### Intracerebroventricular Injection

Central administration of PACAP was performed as described [40]. Briefly, mice were anesthetized and a guide cannula was implanted into the lateral ventricle using a stereotaxic apparatus (Narishige, Japan). After at least eight days for recovery, mice were injected with 20 pmol of PACAP38 (Peptide Institute, Japan; 1 µl min^−1^, total volume 2 µl), under dim red light, 30 min before photic stimulation (100lx) at ZT13; this amount of PACAP38 seems to be within the physiological range [41] and could completely ameliorate the affective disturbance in *Adcyap1* ^−/−^ mice [40]. Control mice were administered with Ringer's solution. At the end of the experiments, successful administration was verified by the infusion of Evance Blue.

### Wheel-Running Activity Rhythms

Adult male mice were individually housed under a 24-hour LD [12 h L (20 or 100lx)∶12 h D] cycle and their activity rhythms were measured for at least eight days using wheel-running apparatuses. Subsequently, mice were transferred to DD conditions and the free running period (τ) was calculated as described [1]. Wheel revolution was quantified using data from three consecutive days, from the sixth day before the start of DD conditions to the fourth day before the start of DD conditions, inclusive, or from the sixth day to the eighth day in DD conditions.

### Slice Preparation, Immunohistochemistry and In Situ Hybridization

LD-entrained mice were transferred to DD conditions and their brains were removed on the 2^nd^ day of DD conditions. To assess ERK phosphorylation, animals were exposed to a light pulse (20 or 100lx) or no pulse (0lx) at CT15, and then perfused with 4% paraformaldehyde in PBS including 1 mM sodium orthovanadate and 0.2 mM phenylmethyl sulfonyl fluoride to inhibit phosphatase activity, at an appropriate time after light stimulation (7.5 min for the 20lx stimulus and 15 min for the 100lx stimulus). We previously confirmed that the level of ERK activation elicited by these stimuli reached a peak at these times. For melanopsin immunostaining, mice were perfused with 4% paraformaldehyde in PBS as described previously [1]. Twenty µm brain sections including the SCN and 5 µm ocular sections were prepared using a cryomicrotome (CM1900, Leica, Germany). Immunohistochemistry and subsequent quantification were undertaken as previously described [1]. Briefly, we used a 1∶1000 dilution of a rabbit anti-phospho-p44/42 Map Kinase (Thr202/Tyr204) antibody (Cell Signaling) for phosphorylated ERK (pERK) or a 1∶1000 dilution of an anti-melanopsin antibody (a gift from Dr. King–Wai Yau), and quantified the number of pERK- or melanopsin-immunoreactive cells in a series of sections (five sections of the SCN and ten sections of the retina per animal). The percentage of melanopsin-expressing cells was calculated as (melanopsin-expressing RGCs/total RGCs)×100.

### Pupillometry

Adult male mice, dark**–**adapted for 1–3 h, were exposed to 1 min of blue light (wavelength: 460–490 nm; light intensities: 20, 100 or 600lx; MVX10, Olympus) without anesthesia. Temporal changes in pupillary responses were archived through an infrared video recorder (Sony, Japan). All tests were conducted between ZT3 and ZT7. Pupil constriction was quantified as described previously [42]. In order to confirm the intrinsic function of the pupillary sphincter, parasympathetic activation by topical administration of carbachol (1 M, Sigma) was also assessed.

### Anterograde Tracing

Mice were anaesthetized with ketamine (60 mg/kg, ip) prior to unilateral intravitreal injection of 2 µl of horseradish peroxidase–labeled wheat germ agglutinin. Subsequently, mice were individually housed for two days and then perfused with 1.25% glutaraldehyde and 0.5% paraformaldehyde. Fifty µm sections were immunostained as described above.

### Reverse Transcription and Real-Time PCR

Adult male mice were killed during ZT14–16 and their left eyes were immediately enucleated under dim red light. Total RNA was extracted using an RNeasy MinElute Cleanup Kit (Qiagen), including DNase I treatment following homogenization in Buffer RLT. One-hundred ng of total RNA was reverse-transcribed using random primers and SuperScript III reverse transcriptase (Invitrogen). Real-time PCR was performed as described previously [43] with some modifications. Briefly, amplification of cDNAs was executed using a Dynamo SYBR Green qPCR kit (Finnzymes) for the PACAP gene and a SYBR Green Real-time PCR Master Mix (Toyobo, Japan) for the other genes. Beta-actin was amplified as an internal control using both PCR kits. The PCR reaction for Thy1 was performed as follows: forward primer 5′–gtcgctctcctgctctcagtcttg–3′, reverse primer 5′ –tcatccttggtggtgaagttggc–3′, 40 cycles of denaturation at 95°C for 5 sec, annealing at 58°C for 5 sec and elongation at 72°C for 20 sec.

### In Situ Hybridization

Brains of mice, kept under DD conditions, were removed at indicated circadian times, then immediately frozen by isopentane, and stored at −80°C until use. Twenty µm brain sections including the SCN and 5 µm ocular sections were prepared using a cryomicrotome (CM1900, Leica, Germany). *In situ* hybridization was performed as described previously [37] using antisense probes containing the coding regions of mouse *Per1* (GenBank accession number AF022992; nucleotides 763–1977), and the mouse Bv8 variant 3 precursor (i.e., *Prok2*; GenBank accession number AF182066; nucleotides 166–414; from a vector gifted by Takeda Pharmaceutical Co. Ltd.).

## Supporting Information

Figure S1 (A) Representative double-plotted actogram during constant dark conditions. Red arrows indicate photic stimulation for 5 or 30 minutes at CT15. Paired red dashed lines represent onset and ending of activity. (B) Photomicrographs showing light-induced ERK phosphorylation, 7.5 minutes (20 lx) or 15 minutes (100 lx) after light stimulation at CT15. Scale bar = 100 µm.
(1.51 MB PDF)
