# Supplementary material for: PACAP-Deficient Mice Exhibit Light Parameter–Dependent Abnormalities on Nonvisual Photoreception and Early Activity Onset
Source: PLoS One. 2010 Feb 18;5(2):e9286. doi: 10.1371/journal.pone.0009286 (PMC2823792; doi:10.1371/journal.pone.0009286)
Supplement: Figure S1 — (A) Representative double-plotted actogram during constant dark conditions. Red arrows indicate photic stimulation for 5 or 30 minutes at CT15. Paired red dashed lines represent onset and ending of activity. (B) Photomicrographs showing light-induced ERK phosphorylation, 7.5 minutes (20 lx) or 15 minutes (100 lx) after light stimulation at CT15. Scale bar = 100 µm. (1.51 MB PDF) [file pone.0009286.s001.pdf]

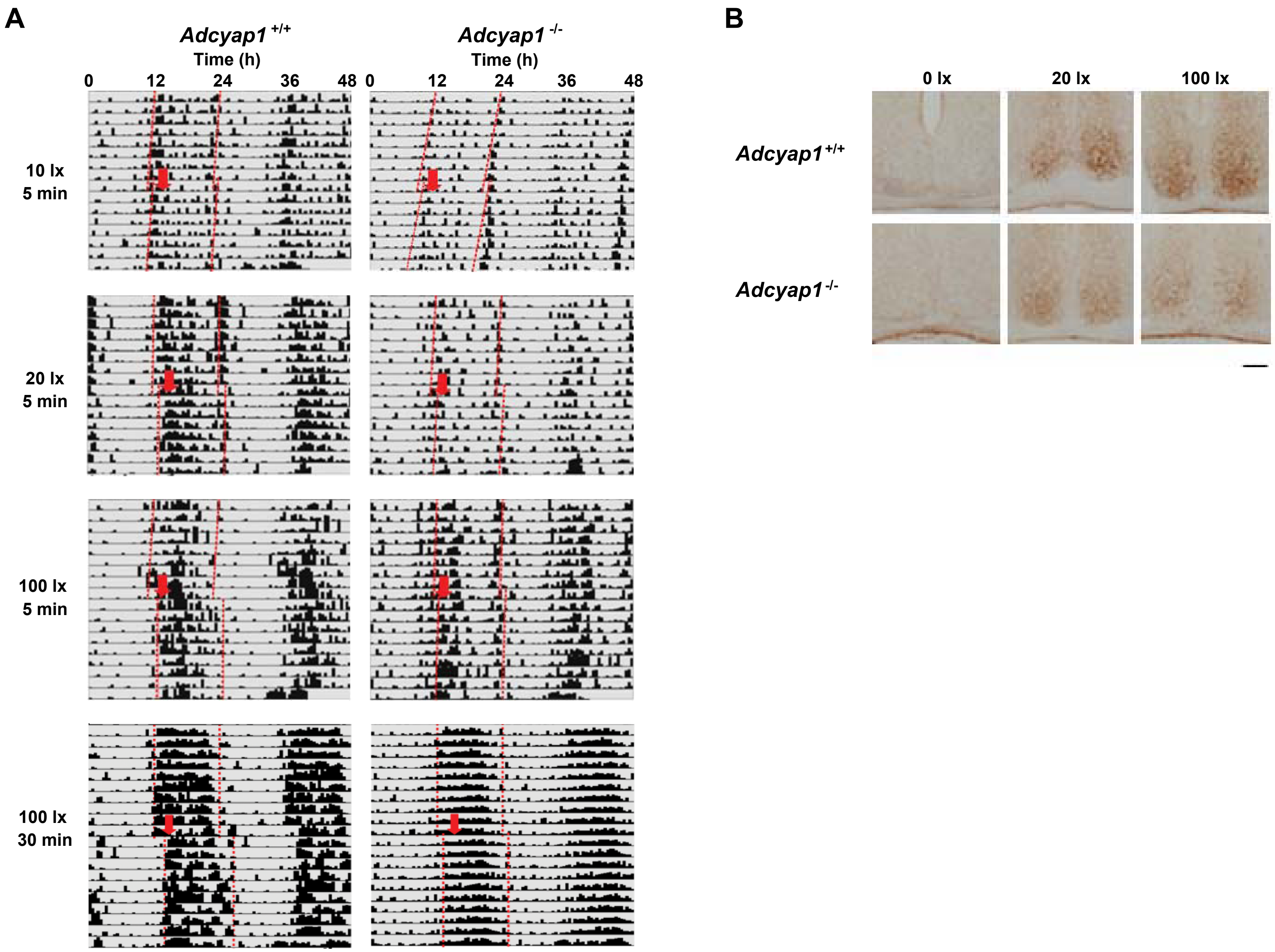

Figure S1. (A) Representative double-plotted actogram during DD conditions. Red arrows indicate photic stimulation for 5 or 30 min at CT15. Paired red dashed lines represent onset and ending of activity. **(B)** Photomicrographs showing light-induced ERK phosphorylation, 7.5 min (20 lx) or 15 min (100 lx) after light stimulation at CT15. Scale bar = 100 μm.
